# Heart Rhythm Insights Into Structural Remodeling in Atrial Tissue: Timed Automata Approach

**DOI:** 10.3389/fphys.2018.01859

**Published:** 2019-01-14

**Authors:** Danuta Makowiec, Joanna Wdowczyk, Zbigniew R. Struzik

**Affiliations:** ^1^Institute of Theoretical Physics and Astrophysics, University of Gdańsk, Gdansk, Poland; ^2^1st Department of Cardiology, Medical University of Gdańsk, Gdansk, Poland; ^3^RIKEN Advanced Center for Computing and Communication, Wako, Japan; ^4^Graduate School of Education, University of Tokyo, Tokyo, Japan

**Keywords:** discrete models of cardiac tissue, cardiac right atrium modeling, heart rate variability, cellular automata, arrhythmia modeling

## Abstract

The heart rhythm of a person following heart transplantation (HTX) is assumed to display an intrinsic cardiac rhythm because it is significantly less influenced by the autonomic nervous system—the main source of heart rate variability in healthy people. Therefore, such a rhythm provides evidence for arrhythmogenic processes developing, usually silently, in the cardiac tissue. A model is proposed to simulate alterations in the cardiac tissue and to observe the effects of these changes on the resulting heart rhythm. The hybrid automata framework used makes it possible to represent reliably and simulate efficiently both the electrophysiology of a cardiac cell and the tissue organization. The curve fitting method used in the design of the hybrid automaton cycle follows the well-recognized physiological phases of the atrial myocyte membrane excitation. Moreover, knowledge of the complex architecture of the right atrium, the ability of the almost free design of intercellular connections makes the automata approach the only one possible. Two particular aspects are investigated: impairment of the impulse transmission between cells and structural changes in intercellular connections. The first aspect models the observed fatigue of cells due to specific cardiac tissue diseases. The second aspect simulates the increase in collagen deposition with aging. Finally, heart rhythms arising from the model are validated with the sinus heart rhythms recorded in HTX patients. The modulation in the impairment of the impulse transmission between cells reveals qualitatively the abnormally high heart rate variability observed in patients living long after HTX.

## 1. Introduction

Biological functionality of an organism is maintained by both the dynamics of individual elements and the network of couplings that arise from the spatial distribution of these elements (Müller et al., 2016). The human heart provides a generic example of such a functionality. The heart tissue has a unique ability to initiate a cardiac action potential and then to spread this impulse from cell to cell, triggering the contraction of the entire heart. Structural modifications of the atrial tissue, caused by heart diseases and/or the normal aging process, influence the performance of the heart contraction. In the following, assuming that the cardiac tissue other than the right atrium works properly, we investigate relations between degradation in right atrial tissue modeled by hybrid automata and the resultant rhythm of heartbeats. From these relations, we expect to find hints about the sources of the unusual heart rate variability (HRV). We also want to learn whether early stages of tissue impairment can be discerned from an analysis of heart rate variability. To this end, we compare rhythms obtained from the model with the rhythms recorded on patients after heart transplantation.

In the case of myocyte electrophysiology, the empirical approach, based on the numerical fitting of the essential myocyte characteristic—the membrane action potential (AP), is particularly substantial. It allows for the straightforward representation of the physiological phases of myocyte excitation by the hybrid automaton states, see e.g., Ye et al. (2005), Ye et al. (2008), and Sloth and Wisniewski (2013). So the important matters of the cellular excitation, for example the variation in the duration of the AP, are directly accessible. However, the main issue of the hybrid approach is its possibility of an almost free design of intercellular relations. The right atrium displays a strong heterogeneous structure. Two clusters of specific and specially organized cells: the sinoatrial node (SAN)—the thin and elongated piece of cardiac tissue built of self-exciting cells, and the atrioventricular node (AVN)—which collects excitations wandering on the atria, and then uniformly transmits them downwards to cause the contraction of ventricles, are crucial elements for the electrophysiology of the heart. The other regions of the atrium, consisting of cardiomyocytes arranged in the crista terminalis, the irregular arrangement of the muscle myofibrils within the pectinate muscle, or Bachmann's bundle leading to the left atrium, impose the complexity in the spread of activation over the atrium (Sánchez-Quintana et al., 2002).

The best way of improving our understanding of such complex and not well-elucidated architecture is through simplified models, such as these based on mechanistic approaches, see for example Nattel et al. (2008), Podziemski and Zebrowski (2013), Nishida and Nattel (2014), and Kharche et al. (2017). Such models allow dealing with many factors acting across multiple temporal and spatial scales. Additionally, it has appeared that continuous models, operating on average electrical properties, are not always satisfactory in capturing details of local heterogeneity of the interacting elements (Gokhale et al., 2017). They are also not adequate in reproducing the diversity of the observations (Chang et al., 2015). Therefore, there are expectations for the development of new techniques, including hybrid models, which allow the combination of discrete and continuous methods, and then allow for the unification of diverse experimental findings (Trayanova, 2014; Mirams et al., 2016). Hybrid automata, which intuitively replace short-lived, transient behaviors with discrete transitions (Henzinger, 1996), offer an attractive approach in this direction. This formalism provides a well-established and efficient tool for simulating real-time systems of complex structure, with elements acting on both continuous and discrete timescales. Therefore the hybrid automata gained considerable attention in systems driving the implantable cardiac medical devices (Jiang et al., 2012; Chilton et al., 2017) or for educational purposes (Spector et al., 2011).

There is also an expectation that the models are helpful in the routine clinical treatment (Trayanova, 2014). In the following, we consider the easy and inexpensive measurement of the heart's electrical activity, namely ECG. Details of ECG curves are inspected by clinicians with the aim of classifying whether a given beat has developed properly, thereby making it possible to refer to it as a sinus (or normal) beat. The length of a normal heartbeat cycle, called RR-interval, changes according to the actual needs of the body for oxygen and other nutrients. In healthy people, this variability is assumed to be driven mainly by the autonomic nervous system and serves an indirect estimation of the autonomic activity (TaskForce, 1996). But people who underwent heart transplantation (HTX) have the nerve pathways providing the autonomic regulation severed during the surgery, so the autonomic regulation of the heart rhythm is provided by hormones circulating in the blood. Consequently, changes in RR-intervals can be considered to represent the intrinsic dynamics of heart performance. Therefore, HTX patients' heart rhythm can be seen as providing a unique opportunity to investigate the relations between structural changes in the heart tissue and resulting alterations in the rhythm of heartbeats.

With current surgical techniques and postoperative immunosuppression, 1-year survival after HTX is about 90%, 5-year survival approximates 70%, and the median survival exceeds 10 years (Thajudeen et al., 2012). There is substantial evidence that heart tissue of an HTX patient is transformed with the passing time. Despite the biological aging process, the permanent intake of immunosuppressive drugs and the complications involved with infections, graft rejection, diabetic and hypertension effects, etc., influence and weaken heart tissue. Also the results of surgery, suture lines, become sources of extra tissue heterogeneity. So long-term HTX recipients have enabled us to track alterations in the dynamics of heartbeats, as has been presented in Makowiec et al. (2016), Wdowczyk et al. (2016), and Wdowczyk et al. (2018).

The objective for our modeling work is to identify in signals of RR-intervals the early warning signs of the underlying tissue remodeling processes. To achieve this goal, we propose a mechanistic model of the right atrium, based on individual cells arranged in a specially designed network. The model is able to generate sequences of RR-interval types specific to the given atrial tissue transformation. So our model allows the investigation of the heart rhythm effects caused by modifications in the intercellular connections, caused by collagen deposition in the cardiac tissue, and also the impact of the overall increase in fatigue of cardiac cells induced by aging or immunosuppressive or other drugs. Thanks to this, the critical dependence between the increase in collagen deposits and the impaired conduction of the impulse and/or the impact of individual cell performance on this conduction could be reliably investigated.

Reconstruction of the short-term dependencies in RR signals observed in HTX patients is the ultimate goal of our modeling. Therefore, the model is validated by nocturnal rhythms of HTX patients recorded by clinical monitoring drug visits to the 1st Department of Cardiology of the Medical University of Gdansk.

The model can be modified and expanded in many straightforward ways. For example, effects of different HTX surgery techniques: biatrial or bicaval, the additional SAN together with the suture line separating the native SAN from the donor atrium (the specificity of biatrial surgery) can be easily simulated. This case is considered in the Appendix. However the specific architecture of nodes, especially the AVN, is left for future model advancement.

The paper is organized as follows. In section 2, the arguments for the chosen method of modeling are given. Section 3 contains a presentation of the methods and materials used. In section 3.1, the model is defined and its numerical motivations are explained. In section 3.2, the signals used for validation of the model are presented. Section 4 contains the results. First we present properties of the model (section 4.1); next properties of real signals are shown (section 4.2). In Section 5 the model is used to explain the high heart rate variability of HTX patients. Finally, in section 6 the model is summarized and we derive conclusions from our findings. In the Appendix, the numerical details of the simulations performed are provided. The source files (dev-cpp project) of the model, as well as the executable file (Windows 7), are accessible from Makowiec (2018).

## 2. Motivation: Discrete vs Continuous Modeling

Mathematical and computational models of cardiac physiology have been an integral component of cardiac electrophysiology since its inception (Mirams et al., 2016). The Luo-Rudy model (Luo and Rudy, 1994; Hund and Rudy, 2004) is assumed to be the primary cardiac cell model reconstructing the real AP. It has its roots in the ion-channel description proposed by Hodgkin and Huxley to model excitation of the neuron axon. The initial model has been enriched in many ways, by adding a much larger number of ion currents and also by including other mechanisms of cellular electrophysiology such as active ions pumps, intercellular compartments for calcium transport, and calcium buffers, see http://www.physiome.org/jsim/models/webmodel/NSR/Luo-Rudy/ or other projects of CellML (Miller et al., 2010). In consequence, the most detailed Luo-Rudy model of the human ventricular myocyte, known now as O'Hara-Rudy (O'Hara et al., 2011), involves 41 state variables in more than 100 differential-algebraic equations; see the latest review of electrophysiological models of the ventricular cell in Carro et al. (2017).

Moreover, myocytes exist in a three-dimensional network built of endothelial cells, forming vascular smooth muscle, and in an abundance of fibroblasts, as well as transient populations of immune cells (Guyton and Hall, 2006). On the scale of groups of cells when the interaction between cells has to be taken into account, the AP is only one aspect of a model reconstructing the cardiac tissue (Trayanova, 2014). Consequently, there are applied FitzHugh-Nagumo (Fitzhugh, 1961; Nagumo et al., 1962) or Fenton-Karma (Fenton and Karma, 1998) simplified phenomenological approaches derived from the biophysical models. What is important is that they are concentrated on capturing the AP shape. To learn more about the variety of dynamics used in modeling cardiac cells, see e.g., Fenton and Cherry (2008).

It is expected that physiological models of cardiac tissue would realize the promise of translational research (Mirams et al., 2016). Namely, they would provide clinical applications for patient-specific approaches such as ablation, cardiac resynchronization and contractility modulation therapies. However, there are still many obstacles to achieving this promise. The reasons are twofold. The first reason is related to variability in cellular activity. It occurs that action potential (AP) shape varies from cell to cell and also in time. The atrial action potential is of a triangular shape and its duration at 90% repolarization (APD_90_) shows variations of between 150 and 500 ms (Fatkin et al., 2007). The second reason comes from the fact that the function of the atria is strongly affected by its anatomical structure (Ho et al., 2002; Ho, 2009). The atria have a number of electrophysiological features which distinguish them from the ventricles and establish their arrhythmic susceptibility (Goette et al., 2016). This partially results from the fact that atrial architecture involves the two nodes: SAN and AVN, and the exits of vessels delivering blood to and from the heart. Both these cardiac nodes: the SAN and the AVN, are sited in the right atrium. The SAN has an elongated shape and its structure of intercellular connections is described as rather loose and free (Sánchez-Quintana et al., 2005). There are two major conduction bundles on the right atrium: crista terminalis (CT), which go along the SAN and lead to the AVN, and pectinate muscle (PM), which in a comb-like fashion spreads throughout the appendage—the large part of the right atrium. Myocytes along the CT are aligned longitudinally, favoring preferential conduction. By contrast, it is not possible to infer the myocyte orientation within other parts of the right atrium (Ho, 2009). Finally, the AVN, which is responsible for the conduction of electrical impulses from the atria to the ventricles, exhibits strong cellular heterogeneity. This heterogeneity leads to the richness of node intrinsic structures and gives rise to a diverse range of macroscopic functions during AVN conduction (Kurian et al., 2010; Hwang et al., 2014).

The complex architecture, together with uncertainty in the AP development, has pushed research toward the probabilistic methods of the Monte Carlo techniques (Mirams et al., 2016), and/or to the cellular automata approach (Lin et al., 2017). However, in any approach, discrete space and discrete time representations of the continuous equations and the types of discrete models used to represent tissue geometry are the numerical protocol requirement; see Clayton et al. (2011) for a discussion on numerical aspects of cardiac models.

### 2.1. Grid Models

In the series of papers of the team from Manchester University, see Li et al. (2008), Li et al. (2014), and Kharche et al. (2017), a mixed approach has been used. The propagation of the AP is considered on the numerical grid made of automata. Eighteen distinct automata and over 3 million elements in a 3D network were applied to capture the diversity of the right atrium tissue (Li et al., 2014). Thanks to that they manage to successfully simulate a wide range of physiologically known facts about the atrium electrophysiology.

In Podziemski and Zebrowski (2013) an even simpler model of the atria has been proposed. It follows the coupled map lattice approach, namely, a two-dimensional square plane is used to represent the geometry. The implementation of the model allows putting non-conductive regions inside the model matrix to simulate anatomical details necessary for the arrhythmia to develop. It consists of 100 × 100 simulation cells divided into dedicated regions: the SAN, AVN and regions of normal atrial conductive tissue in between. However, for cellular dynamics and intercellular interactions, FitzHugh-Nagumo and Fenton-Karma sets of dynamics for the ion-channels activity of cardiac cell were used.

### 2.2. Cellular Automata Approach

In the classical Greenberg-Hastings cellular automata model of excitation, three-state (denoted: active *A*, firing *F* and refractory *R*) automata are placed on a regular lattice (Greenberg and Hastings, 1978). Each automaton in the active state becomes firing if the number of firing neighbors exceeds a certain threshold. Then an automaton moves from firing to the refractory state, to return back to its original state of being active in a few time steps. The emergence and disappearance of dynamical objects typical of cardiac arrhythmia episodes—spirals—have been shown with heterogeneous Greenberg-Hastings automata (Bub et al., 2005). Also, atrial fibrillation—the most commonly known atrial arrhythmia, as the effect of impaired functioning of cells and/or fibrosis, has also been reproduced with this approach (Christensen et al., 2015; Manani et al., 2016).

### 2.3. Hybrid Automata Approach

As electrophysiological processes in a myocyte are driven by a set of thresholds for opening and closing paths for ion movements across the cellular membrane, myocytes can be reliably represented by hybrid automata where the continuous oscillatory dynamics is mixed with transitions between automaton states (Ye et al., 2006; Sloth and Wisniewski, 2013). It has become apparent that such hybrid automata can very accurately reproduce the membrane potential (Ye et al., 2008). Then, a stochastic network of timed automata (Ye et al., 2008; Bartocci et al., 2010) makes it possible to model directly ionic currents and, as follows, wavefront propagation with respect to the atrial anatomy.

In a series of papers (Ye et al., 2005, 2006, 2008) one can find a systematic comparison of the results obtained from a lattice of nonlinear differential equations commonly used to represent the myocyte ion channel dynamics, to the hybrid automata approach in which short-lived, transient behaviors are replaced by discrete transitions. By this approach, one achieves the solution efficiently, namely about ten times faster than with the approach which uses the complete set of differential equations (Ye et al., 2005). Furthermore, it has been found that simpler automata, called timed automata, can effectively capture details of the biochemical dynamics of a myocyte and specificity of cell-to-cell interactions (Bartocci et al., 2010; Jiang et al., 2012).

All of the above described modern computer techniques provide a framework in which their computational capabilities, possible problems, can be systematically verified and justified.

## 3. Materials and Methods

### 3.1. Timed Automata Model of the Right Atrium

#### 3.1.1. A Timed Automaton

A timed automaton according to Ye et al. (2008) and Jiang et al. (2012) refers to:

(a1) a graph *G*(*V, E*) where the set of vertices *V* represents the system states (often denoted as *S*) and the set of edges *E* (often denoted as $\overset{\Sigma }{\to }$), which contains labeled transitions between the states;
(a2) a finite set *X* = {*x*_1_, …, *x*_*n*_} of real variables called clocks, because all these variables evolve synchronously, either increasing its value by 1 in each time step or resetting its value to 0 in the case of transitions;
(a3) a family of clock invariants for each state: conditions (formally, predicates) on the clock values for a given state;
(a4) a family of transition guards for each edge: constraints (formally, predicates) on the values of the clocks which have to be true for a transition represented by a given edge.

Accordingly, the clocks record the passage of global time in the system, what synchronizes elements in the system. Transitions are controlled by the state invariants which preserve the system in a given state and clock constraints which push transitions between the states. Thanks to these double time guards, each automaton can pursue its individual life.

Often, a timed automaton is considered to be a transition system with two types of transitions:

T1: time transitions by any time interval δ > 0 when only time progresses but the automaton state is not changed;
T2: state transitions when the automaton state changes. T2 is usually accompanied by a time reset.

**Figure 1:**
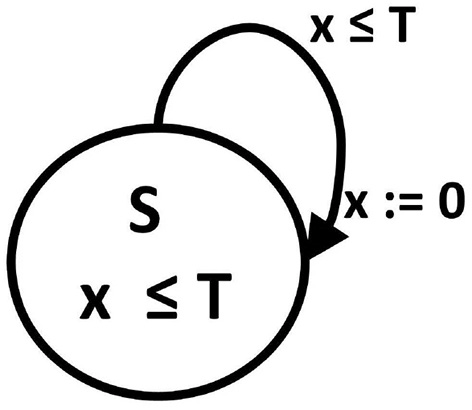
A phase oscillator as an oscillator timed automaton. A digraph of state transitions: *S* is a state name, *T* is the cycle length which determines the state invariant and transition guard. *x* is a clock variable, the value of which advances by one in each time step, and which is reset to 0 after reaching the threshold *T* value.

Specifically, a phase oscillator, i.e., oscillator characterized by the phases only, can be represented by an oscillator timed automaton. This automaton is built on (a1) graph consisting of one state *S* and one T1 -type edge, (a2) with one clock variable *x*, (a3) for which the invariant for the *S* state means *x* ≤ *T* where *T* is the oscillator period, and (a4) the transition occurs when *x* ≥ *T*. The transition means resetting the clock value to 0. Such oscillators are basic elements in the Peskin model (Peskin, 1975) and Kuramoto model (Kuramoto, 2003), with which the collective synchronization of pulse-coupled oscillators has been successfully investigated. It is easy to notice that an angular phase of an oscillator is replaced in the timed automaton by the time elapsed in a given period, see Figure 1.

#### 3.1.2. A Cell—Myocyte Electrophysiology as Oscillator Timed Automaton

Formally, we will be considering the following automaton, further referred to as a cell (Ye et al., 2006; Bartocci et al., 2010): *A cell* is an oscillator timed automaton represented by a graph $G\left(S,\overset{\Sigma }{\to }\right)$ of states *S* and labeled transitions $\overset{\Sigma }{\to }$, a clock set *X* and sets of state invariants *inv* and transition guards Θ, where

(a1) *S* = {*F, R, A*} is a three-state space, and $\overset{\Sigma }{\to }=\left\{\left(A\overset{\text{fire}}{\underset{}{\to }}F\right),\;\left(F\overset{\text{refract}}{\underset{}{\to }}R\right),\;\left(R\overset{\text{activate}}{\underset{}{\to }}A\right)\right\}$ contains three transitions between these states, the labels of transitions are *fire, refract* and *activate*, respectively;
(a2) a clock set *X* consists of one variable *x*;
(a3) an invariant *inv*() is defined for each state as:
  $$\begin{array}{l} inv\left(F\right)=\left\{x\leq f\right\},\\ inv\left(R\right)=\left\{x\leq r\right\},\\ inv\left(A\right)=\left\{x\leq a\right\}, \end{array}$$
  for some real and positive numbers *f, r* and *a*;
(a4) a clock guard Θ() is defined for each transition:
  $$\begin{array}{l} \Theta (F\overset{\text{refract}}{\to }R)=\{x\geq f\},\\ \Theta (R\overset{\text{activate}}{\to }A)=\{x\geq r\},\\ \;\Theta (A\overset{\text{fire}}{\to }F)=\{x\geq a\}, \end{array}$$
  for the real and positive numbers *f, r* and *a* introduced in invariants. Each transition is followed by the clock reset to 0.

Thus the actual properties of a cell can be described by a pair (*s, x*) of values representing a state *s* ∈ *S* and a time count *x*, elapsing with the time steps that a cell stays in *s*. Then, the intrinsic cell cycle can be rewritten as the following transition system:

T1: a time pass move by δ > 0
  (1) $$(s,x)\overset{\delta >0}{\to }(s,x+\delta )\text{ only if }x+\delta \leq inv(s);$$
T2: an instantaneous transition from *s* to *s*′ state with time reset:
  (2) $$(s,x)\overset{(s\overset{a}{\to }s^{\prime })\in \overset{\Sigma }{\to }}{\to }(s^{\prime },0)\text{ if }x\geq \Theta (s\to s^{\prime }).$$

Both rules T1 and T2 are deterministic and therefore the resulting evolution will be called deterministic. We say that the evolution is stochastic if a cell performs T2 with the following constraint:

ST2: a cell can refuse the *fire* transition with probability *p*_refuse_ despite the count variable crossing the invariant value *a*.

**Figure 2:**
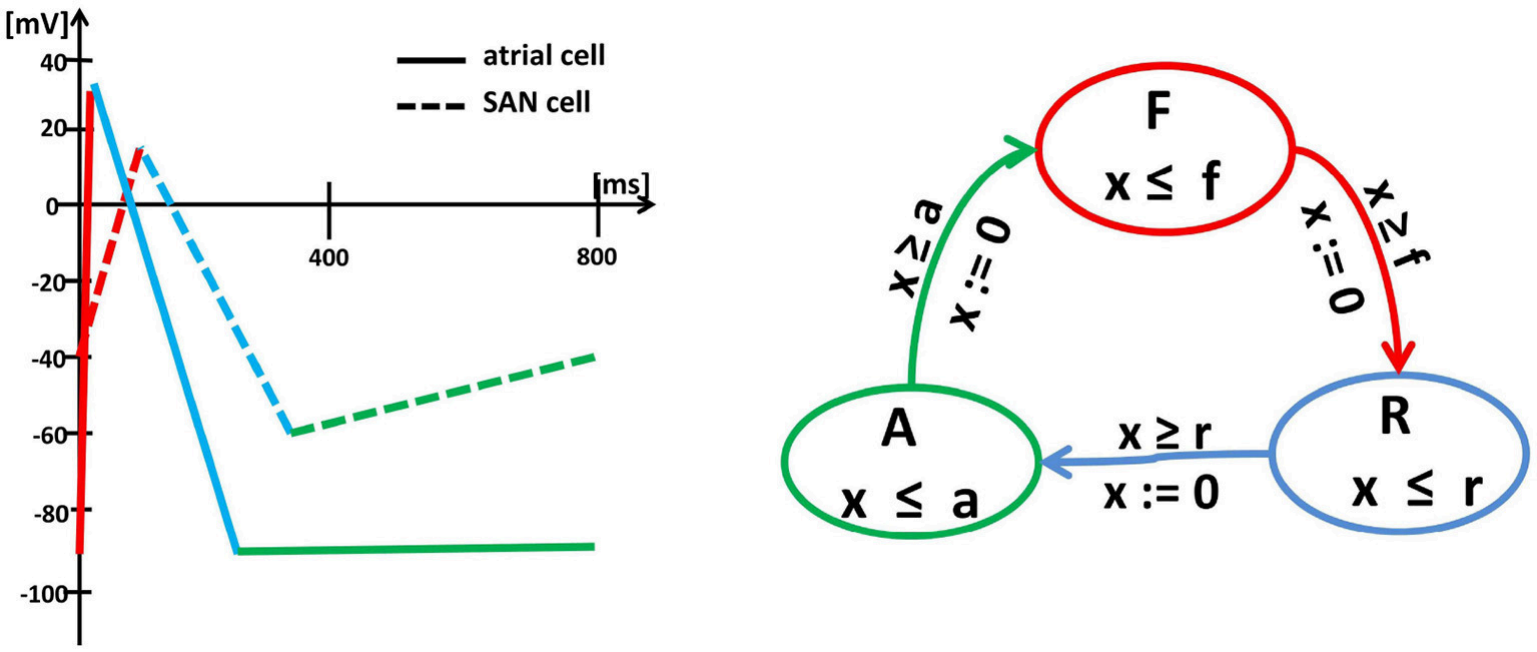
*Left:* Schema (piecewise linearization) of the myocyte membrane action potential in a typical SAN cell (dashed lines) and a typical atrial cell (solid lines). Changes in the membrane potential result from the activity of specific ionic currents. In an atrial cell, the rapid depolarization (red) results from the activation of the inward Na^+^ currents. The length of the action potential is determined by the balance between the inward currents (mainly Ca^2+^, which tend to keep the cell depolarized) and the outward currents (mainly K^+^, which tend to repolarize it) during the action potential plateau (blue). Then, the membrane potential returns to the initial level (green) and remains there until the next excitation. In a pacemaker cell, the membrane depolarization results from the inward Ca^2+^ (red), which is then pacified by the outward K^+^ (blue). Moreover, for a SAN cell, after reaching the lowest membrane potential again, the *funny* inward current is activated, which slowly leads the cell to the self-excitation. *Right:* Graph of the oscillator timed automaton of three states **F**, **R** and **A** and cyclic transitions between them. The graph displays the intrinsic cycle of an isolated SAN cell. The states are related to linear pieces of the membrane action potential, i.e., **F** corresponds to rapid currents of Na^+^(atrial cell) or Ca^2+^(SAN cell) while **R** to the action of Ca^2+^ and K^+^. The transitions between states **F** , **R** and **A** are limited by clock guards **f** , **r**, and **a**. Note, that an atrial cell and a SAN cell can be represented by the same timed automaton, but with different clock invariants and guards.

The approach formulated above follows the so-called empirical approach (Sloth and Wisniewski, 2013), which means that it results from the combined analysis of the shape of curves obtained from the numerical integration of differential equations describing the action potential evolution and from the empirical observations; see Figure 2. Such an approach preserves the solution accuracy in about ten times shorter computational time; see Ye et al. (2005).

#### 3.1.3. A Stochastic Network of Cells

**Figure 3:**
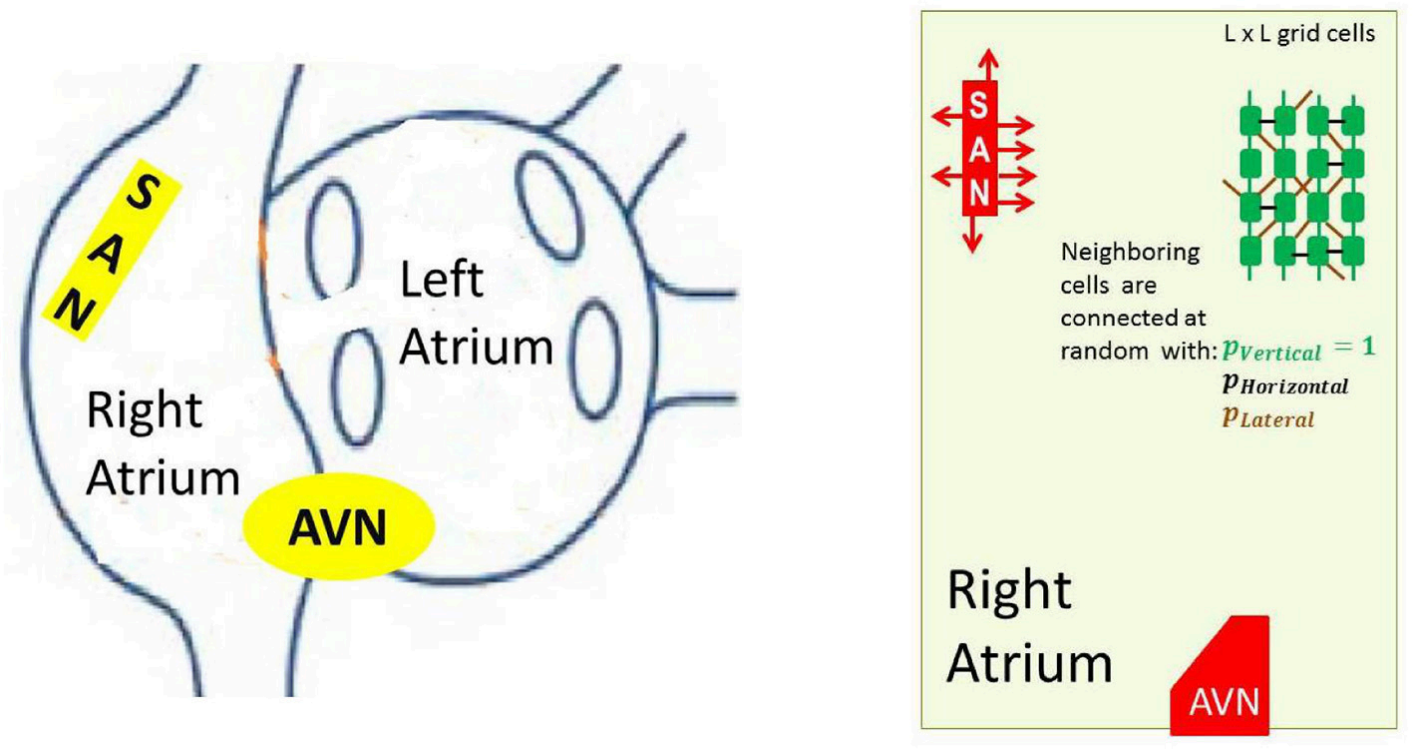
*Left:* A schema of atria to show the anatomical arrangement of right and left atrium, and the SAN and the AVN. *Right:* The frame within which our model works. Arrows around the SAN display discrete connections between the SAN and atrial cells— the SAN-atrial pathways. Additionally a cellular grid with stochastic intercellular connections driven by parameters *p*_*V*_, *p*_*H*_ and *p*_*L*_ is shown.

In the reconstruction of the sinus beats and the right atrium performance, specific elements, like the SAN and AVN, have to be included. The cellular grid considered by us, see Figure 3, follows commonly assumed atria architecture; see e.g., Nattel et al. (2008), Nishida and Nattel (2014), and Goette et al. (2016).

In particular, to mimic the elongated shape of a real node, the SAN is represented as a group of cells in a rectangular shape. The SAN cells cannot read signals from outside; they only send signals to the atrium, which is in agreement with the known physiological facts about the SAN (Sánchez-Quintana et al., 2005). Moreover, we consider limitations in connections between the SAN and the atrial cells by assuming that only half of randomly chosen bordering SAN cells are able to transmit excitations to the atrium. The AVN is assumed here to be a passive element. A small group of cells located in the middle of the bottom boundary collects activation of the surrounding atrial cells.

The formal specifications of the stochastic network N representation of the right atrium architecture is as follows:

(i) cells with randomly set refractory time threshold
  (3) $$\begin{array}{r} r=r_{0}+r_{\text{noise}}\xi \end{array}$$
  for ξ ∈ *U*[0, 1] a uniformly distributed random variable, are arranged in the vertices of a regular square lattice of linear size *L*;
(ii) each automaton, except those of the boundary, is connected with the probability *p*_*V*_ = 1 to its vertical neighbors N and S, with probability *p*_*H*_ to its horizontal neighbors E and W, and with probability *p*_*L*_ to its lateral neighbors NE, NW, SW and SE (lettering used follows geographic map directions); the resulting mean number of neighbors is
  (4) $$\begin{array}{r} {\text{mean}}_{J}O\left(J\right)=2\left(p_{V}+p_{H}\right)+4p_{L}, \end{array}$$
  where O(J) denotes a set of cells neighboring the cell J∈N;
(iii) the SAN is represented as a compact group of cells of a rectangular shape located in the left upper part of the lattice, the SAN vertical connections between cells are stochastically free, namely *p*_*V*_ = *p*_*H*_ = *p*_*L*_;
(iv) the AVN is a small compact group of cells located in the middle of the bottom boundary. To enhance the role of AVN as an organ, we assume the AVN excitation only if simultaneously a large part of AVN cells (here, 1/3) is excited.

#### 3.1.4. Interactions Between Cells

It is also known that interactions between myocytes influence the performance of the cellular cycle (Anumonwo et al., 1991; Coster and Celler, 2003). It is known that earlier excitation of a myocyte or its prolonged stay in the refractory state can result from a high excitation level of the surrounding myocytes. The following transition scheme remains in agreement with the known physiology.

For each cell J∈N, let the expression [*s*_*J*_ = = *F*] give the result of the test if the state of the cell *J* is *F*. Then

T1^*^
  (5) $$\left(R,x)\overset{\Sigma_{J^{\prime }\in O(J)}[s_{J^{\prime }}==F]>N_{R}}{\to }(R,\left\lfloor \frac{x}{2}\right\rfloor \right)$$
  with *N*_*R*_ being the threshold for effectiveness of the external stimulation when a cell is in *R* state.
T2^*^
  (6) $$\left(A,x)\overset{\Sigma_{J^{\prime }\in O(J)}[s_{J^{\prime }}==F]>N_{F}}{\to }(F,0\right)$$
  with *N*_*F*_ being the threshold for effectiveness of the external stimulation when a cell is *A* state.

The effects of the above rules on the synchronization in a complex network of oscillator timed automata are discussed in detail in Makowiec (2014).

### 3.2. Short-Term Heart Rate Variability by Probability Matrix of Patterns

Twenty-four-hour ECG Holter measurements were collected from the patients of the HTX group within the standard clinical observation procedure. The following exclusion criteria were applied: a history of pacemaker implantation, non-sinus rhythm, clinically unstable condition, and unwillingness to participate in Holter monitoring. At the time of the measurements, the patients were in good physical condition without echocardiographic signs of acute rejection, heart failure or left ventricular dysfunction. We also excluded ECG Holter recordings with frequent ventricular and supraventricular arrhythmia and with more than 10% artifacts. All the subjects gave their written, informed consent, which was approved by the Ethics Committee of the Medical University of Gdańsk.

The recordings were analyzed on a Del Mar Reynolds system (Spacelabs Healthcare, United States). The sampling rate of ECG was 128 Hz, which led to 8 ms accuracy in the identification of R-peaks of the QRS complex. The quality of the ECG recordings and accuracy of R-peak detection were verified by visual inspection by experienced cardiologists. All normal beats were carefully annotated so that only normal sinus rhythms were considered in our investigations. The period of nocturnal rest was discerned individually, in each recording separately, according to the appearance of consecutive hours with a low heart rate. Then each signal was edited to preserve RR-intervals between normal-to-normal beats only. Short segments with artifacts or not normal beats (less than 5 events) were substituted by the medians estimated from the last seven normal beats. Longer segments with wrong data were deleted, which was annotated correspondingly. Finally, signals consisting of twenty thousand beats were selected, starting at the beginning of the nocturnal period.

Having extracted successive RR-intervals: *RR*_0_, *RR*_1_, …, *RR*_*N*_ the sequence of differences between consecutive RR-intervals, was derived: δ*RR*_1_, …, δ*RR*_*N*_ with δ*RR*_*k*_ = *RR*_*k*_ − *RR*_*k*−1_ for *k* = 1, …, *N*. Then, for each signal there was estimated a probability for two-event patterns (Δ*RR*(*i*), Δ*RR*(*j*)) with Δ*RR*(*i*) and Δ*RR*(*j*) describing the two events subsequent in the signal. The matrix of these probabilities *P*(Δ*RR*(*i*), Δ*RR*(*j*)) has been found to represent in the best way the extraordinary (erratic) events in the heart rhythms (Makowiec et al., 2016; Wdowczyk et al., 2018).

## 4. Results

### 4.1. Model Characteristics From Simulations

All the practical details of our simulations are listed in the Appendix. Below we review the observed results focusing on the critical dependence emerging in a system when the model parameters are modified. We concentrate on parameters which influence the stationary state organization and have an important physiological meaning. Accordingly, we focus on the following aspects:

- The rule ST2 of the cell definition given in section 3.1.2, which allows any cell with probability *p*_refuse_ to refuse for the excitation, can be interpreted as the mean ability of the atrial tissue to respond to stimulation. In particular, the tissue impaired by cardiac disease, aging and/or drugs could be considered to display this kind of tissue fatigue. Consequently, *p*_refuse_ > 0 refers to the impaired tissue.
- The relation between the density of intercellular connections and the fibrosis of the cardiac tissue is commonly accepted. In the following, assuming that all atrial cells are vertically connected, we concentrate on the density of the non-vertical connections, assuming that lateral and horizontal connections are equally probable, *p*_*L*_ = *p*_*H*_.
- Additionally, we test the effect of the variability in the APD of cardiac cells by considering the stochastic setting of the length of the refractory state of atrial cells. This dependence is driven by *r*_noise_ of Equation (3).

All other parameters are kept constant. For these reasons the results presented are quantitative only. The critical values presented below are dependent on the settled values of other parameters as, for example, they depend on the ratio of AVN cells excited to count the excitation of the AVN; see (iv) of section 2.4.

#### 4.1.1. Limit States Classification

The heart rhythm is denoted from time steps between subsequent AVN excitations. The wavefront reaching the AVN must be consistent enough to excite the AVN. As we assume that from AVN the impulse propagates unperturbed, we can consider a series of subsequent step distances between the AVN excitations to be comparable to the sequence of heart contractions *RR*(*i*).

**Figure 4:**
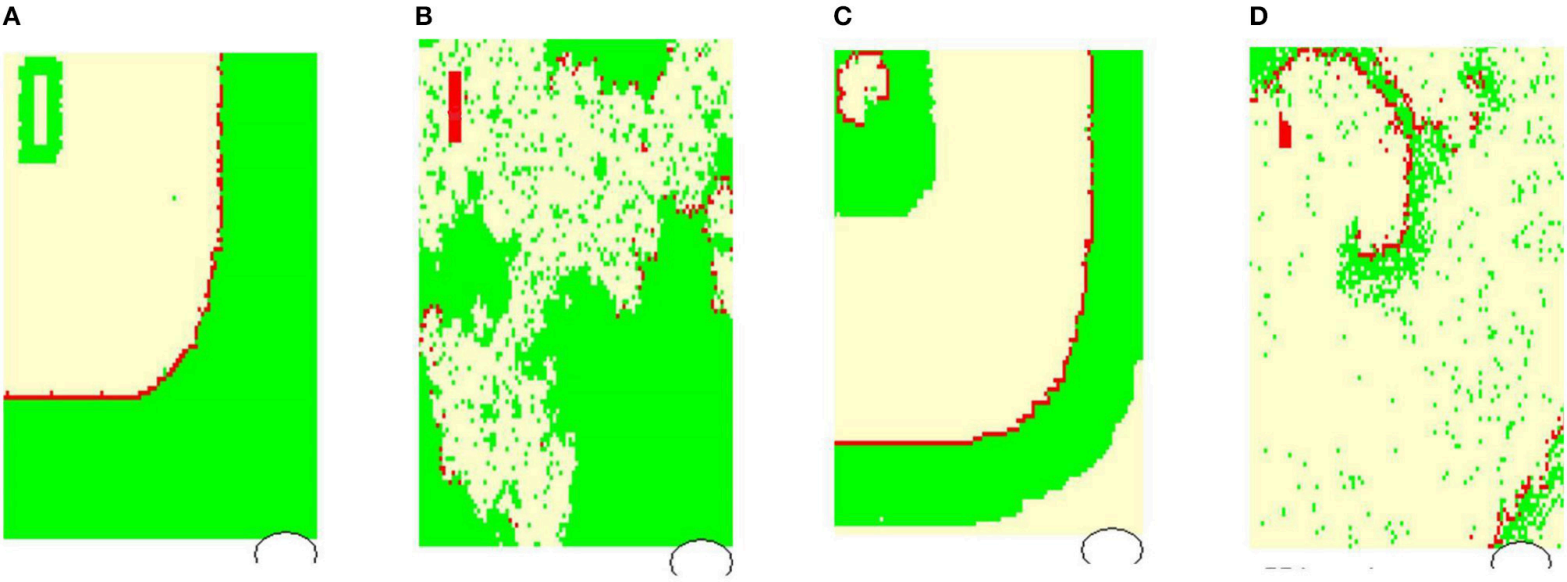
Typical wavefronts observed in simulations for *p*_*L*_ = *p*_*H*_ = 0.50, *r*_noise_ = 0, *p*_refuse_ = 0%: **(A)** *normal state* wavefront; **(B)** loss of the regular wavefront due to high level of *p*_refuse_ = 50%; **(C)** *SAN arrhythmia* due to self-sustained excitation resulting from a path around the SAN; **(D)** fibrillation—sustained rotating waves of origin different from the SAN, which result from the rapid switch from the lost front state **(B)** with *p*_refuse_ = 50% to *p*_refuse_ = 0%. The red color marks cells in *F*, white in *R*, and green in *A*.

**Figure 5:**
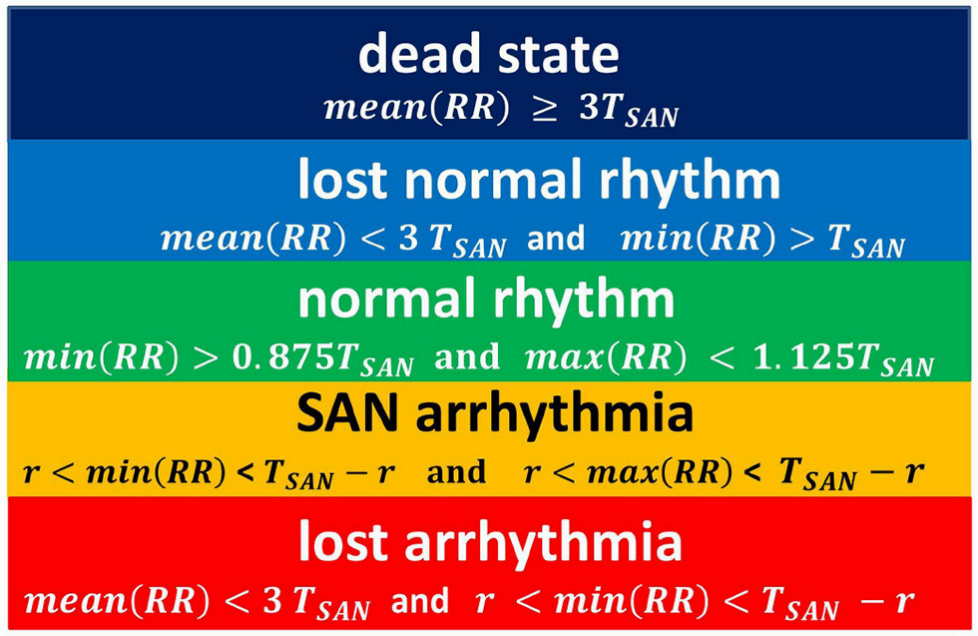
The rules for classification of states observed in simulations. The classification is driven by properties of a series *RR*(*i*).

Figure 4 shows typical limit states of the excitatory front observed under certain model parameters. In Figure 5 we propose the classification of these states according to properties of the *RR*(*i*) signals.

This proposition for the classification of limit states is idealistic in the sense that it refers to the length of the typical RR-interval observed in a given simulation and stability of the rhythm. Basically, we compare simulation results to the human RR-intervals variability, see TaskForce (1996). Namely, in a healthy man, an increment Δ*RR*(*i*) greater than 200 ms is assumed as physiologically questionable. Considering that 800 ms represents the mean RR-interval, one could find 200ms/800ms = 0.25 as the estimates for the variability of a healthy man's rhythm. Below we will use this relation. Also, we give further arguments for our classification rules.

In Figure 4A, the line of the wavefront propagates from the SAN to the AVN. Each SAN excitation results in the formation of the front line. At each time step, only one front line propagates over the system. So each AVN excitation follows a SAN excitation. The heart beats at the frequency of the SAN excitation, i.e., mean(RR(i)) approximates *T*_*SAN*_ the period of SAN cells self-organized excitation *T*_SAN_ = *f*_SAN_+*r*_SAN_+*a*_SAN_. Following the facts mentioned above about the variability of the normal human rhythm, we expect the normal rhythm (the sinus rhythm) to satisfy the following condition:

(7) $$\begin{array}{r} \underset{i}{\max}RR\left(i\right)-\underset{i}{\min}RR\left(i\right)<0.25T_{SAN}. \end{array}$$

When the ratio of cells refusing the excitation becomes large enough, namely *p*_refuse_ increases, the loss of the line of the wavefront is observed; see Figure 4B. Some SAN excitations do not reach the AVN; they are missed. Eventually, at a very high level of *p*_refuse_, there are many waves wandering over the tissue. Only a few of them reach the AVN. The AVN excitation rhythm is almost absent, or if it occurs, it is very different from the rhythm of the SAN. Such a rhythm will be called dead. In the following, we propose to consider a state as a dead state if the mean(RR) is three times larger than the SAN period. This denotes that more 2/3 SAN excitations are lost.

Due to the special arrangement between SAN and atrial cells, it can happen that one observes two fronts propagating over the network; see Figure 4C. The first front comes from the SAN excitation; the second front results from the sustained activity circulation around the SAN. This second front is a manifestation of self-organization between the cell dynamics and the network structure. In consequence, two fronts reach the AVN within one SAN normal period. Hence, the AVN excitations are doubled when compared to the SAN excitations. The minimal *RR*(*i*) distance depends on the length of the atrial cell refractory *r*. We will call such a rhythm the SAN-atrial re-entry rhythm or, in short, SAN arrhythmia.

Finally, in Figure 4D, we present a state of fibrillation, i.e., the emergence of self-supporting rotating waves acting among atrial cells independently of the SAN. Such states can develop when *p*_refuse_ is not stable, i.e., when after a period when *p*_refuse_ is high enough, the system recovers a low value of *p*_refuse_.

#### 4.1.2. Results of Deterministic Dynamics: *p*_refuse_ = 0

The deterministic evolution results from rules T2 and T2^*^ applied with *p*_refuse_ = 0. The only source of stochasticity is the random network organization and diversity of cells. The network organization includes a density of intercellular lateral and horizontal connections quantified by *p*_*H*_ = *p*_*L*_. Diversity of cells, governed by *r*_noise_, denotes stochastic variety in the action potential duration driven by the formula *r* = *r*_0_ + *r*_noise_ξ for ξ ∈ *U*[0, 1].

**Figure 6:**
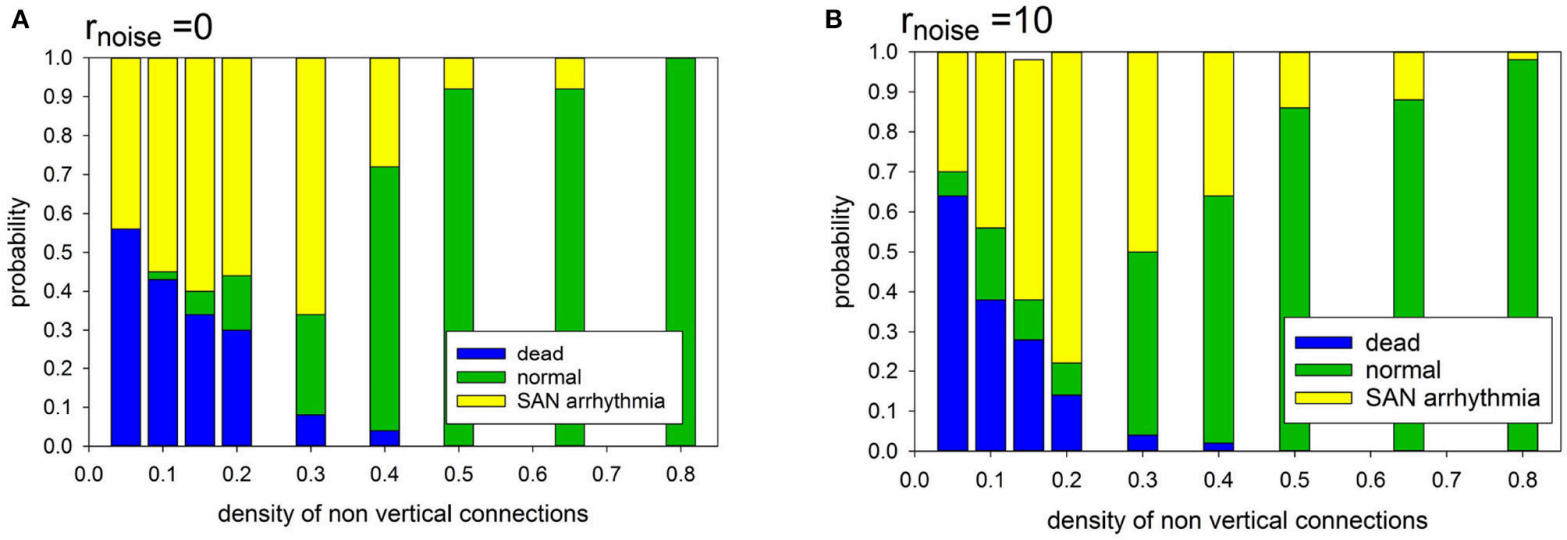
Statistics of limit states in case *p*_refuse_ = 0.0, for different density of transversal connections and for different cell organizations: **(A)** cells with identical APD; **(B)** cells with stochastically distributed APD at *r*_noise_ = 10.

In the case of the deterministic evolution, the following three limit states develop: normal, SAN arrhythmia and dead. However, the proportions between them depend on the specificity of the network organization and on the presence or absence of diversity among cells. In Figure 6, we show the observed relations between the states.

It is noticeable that in the case of a low intercellular density of connections two limit states dominate: SAN arrhythmia and dead state. The normal rhythm occurs negligibly rarely. The sharp transition to occurrence of more than 50% of normal states takes place if *p*_*H*_ = *p*_*L*_ ≥ 0.40.

Note that when the atrial cells are stochastically heterogeneous due to the random spread of action potential duration (APD), namely *r*_noise_ = 10 then normal states occur more frequently at the low intercellular density of connections. Hence, differentiation of cells enhances the functionality of the system at low densities of intercellular connections. However, in the case of high density, this observation is not true. The participation of states with SAN arrhythmia is higher than in a system of identical cells. In the following, we have considered *r*_noise_ = 10 which allows us to enter about 20% uniform variation in the length of the *R* state.

#### 4.1.3. Effects of Dynamical Stochasticity: *p*_refuse_ > 0

As could be expected, with switching *p*_refuse_ > 0, two families of lost rhythm occur. These new states can be described as missed-beat states. There are two classes of these states. The first class arises from the sinus rhythm, in which some SAN excitations are lost before reaching the AVN. The second class consists of rhythms related to arrhythmia states, as these rhythms display the presence of RR-intervals significantly shorter than the period of the SAN. They partially relate to SAN arrhythmia with lost beats but also they are a manifestation of an other self-organization of cellular excitations than from the SAN, namely fibrillation.

**Figure 7:**
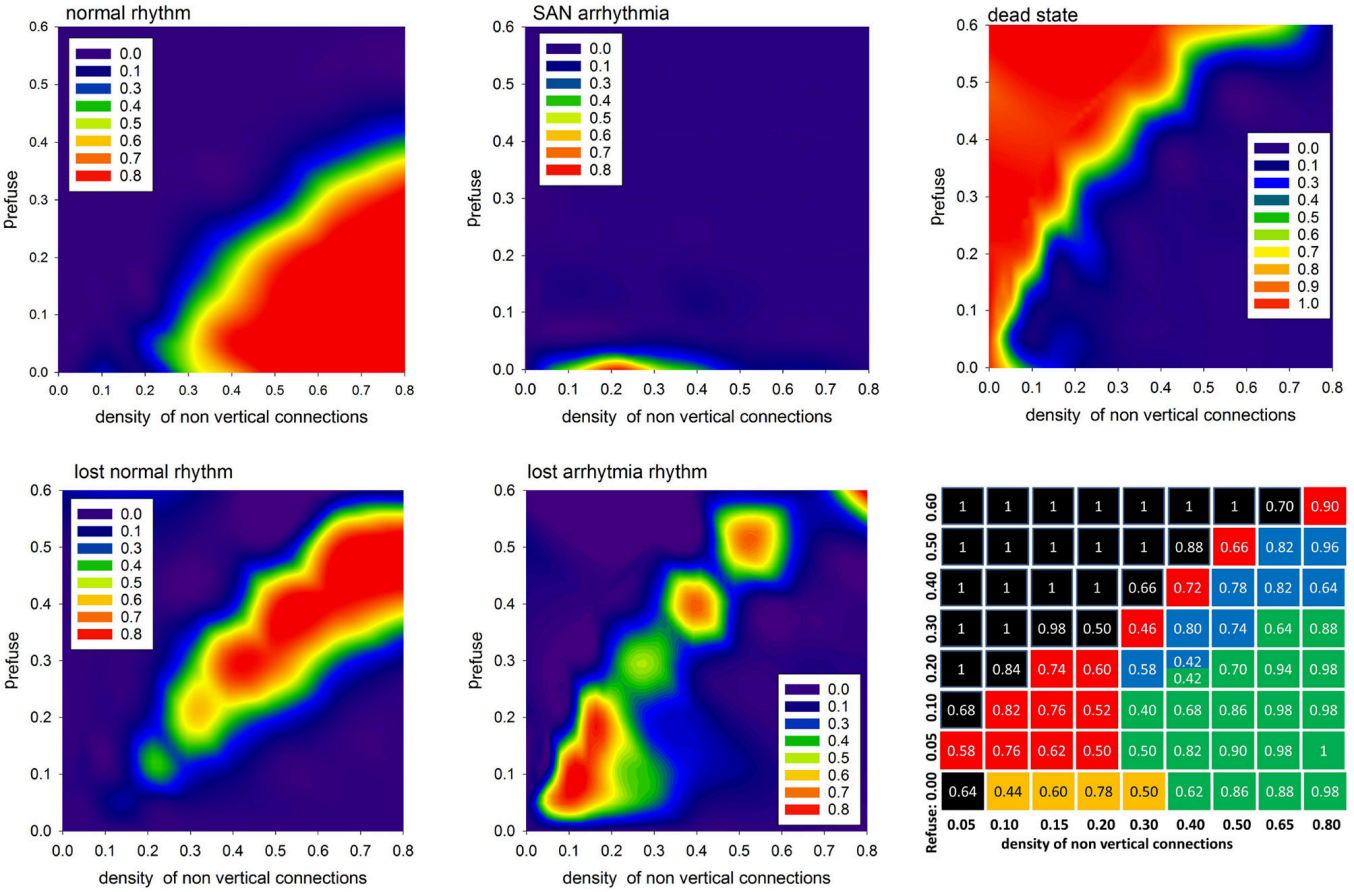
The contour plots displaying probability to observe the given limit state. The last bottom panel provides probability values of the most probable state for the given *p*_*L*_ = *p*_*H*_ and *p*_refuse_. The values are in case the APD is set stochastically with *r*_noise_ = 10.

In Figure 7 we show the probabilities to find the system in the given states: normal, SAN arrhythmia, lost normal, lost arrhythmia, and dead, according to increasing density of non-ventricular connections *p*_*L*_ = *p*_*H*_ and increasing fatigue of the cells driven by *p*_refuse_ > 0. We see that the areas of high probability of the occurrence of a particular state have rather sharp borders. To support this observation, we present the table with probability values of the most probable states for a given model parameter.

One can see that the state space of the model parameters (*p*_*H*_ = *p*_*L*_, *p*_refuse_) is critically divided into regions of substantial domination of a particular state. It is important to repeat that the statistics presented here strongly depend on what we mean by AVN excitation. In particular, the results shown in Figure 7 were obtained assuming that the AVN excitation takes place when three of eight AVN cells are simultaneously excited.

#### 4.1.4. Velocity of Excitation

The velocity of the impulse propagation is one of the most important characterizations for quantification of the cardiac tissue transformation caused by collagen deposits and/or cell impairment. In the case of timed automata, this property is clocked by synchronized steps of the whole system. However, propagation of the wavefront resulting from the first SAN excitation can be considered to display the spatial organization of the actual system. Because all the cells are vertically connected, this first front reaches the bottom border in the fastest way possible with the actual arrangement of cellular dynamics, i.e., the value of *p*_refuse_. Therefore, one can assume that the tissue performance for the propagating impulse can be estimated by the difference between the time steps in which this first front reaches the AVN and the time steps in which this front reaches the bottom border. This means that the larger this difference is, the smaller the velocity of the front propagation is.

**Figure 8:**
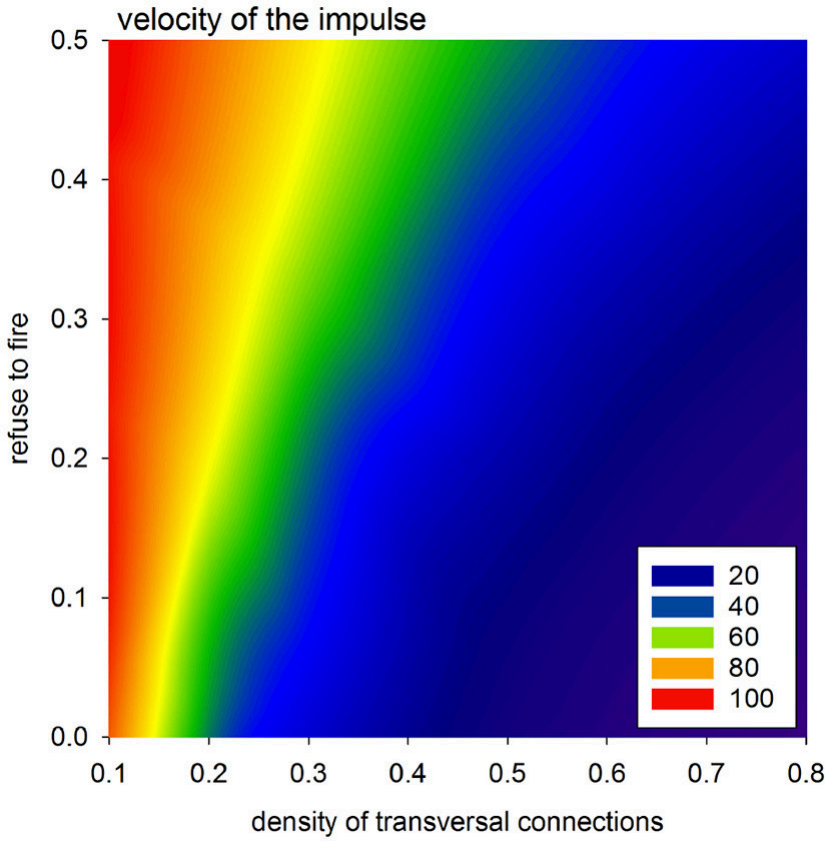
Velocity of the spread of the impulse according to the difference in transmission signals with a perfect network organization and otherwise.

In Figure 8, we present mean values of this difference, based on the simulations performed with a uniform stochastic APD distribution due to *r*_noise_ = 10. One can see that a high density of transversal connection guarantees efficient impulse propagation, even in conditions when a large number of cells refuse excitation. It is noticeable also that the density of transversal connections *p*_*H*_ = *p*_*L*_ = 0.5 separates a stable system organization from a possible unstable system organization.

### 4.2. Short-Term Variability for Long-Term HTX Patients

The probability matrix *P*(Δ*RR*(*i*), Δ*RR*(*j*)) provides estimates for the short-term dynamical dependence in the normal-to-normal heartbeat RR-increments (Makowiec et al., 2015). These probability matrices, shown as contour plots, in a compact way display the abundance of acceleration (negative Δ*RR*(*i*)) and deceleration (positive Δ*RR*(*i*)) patterns.

**Figure 9:**
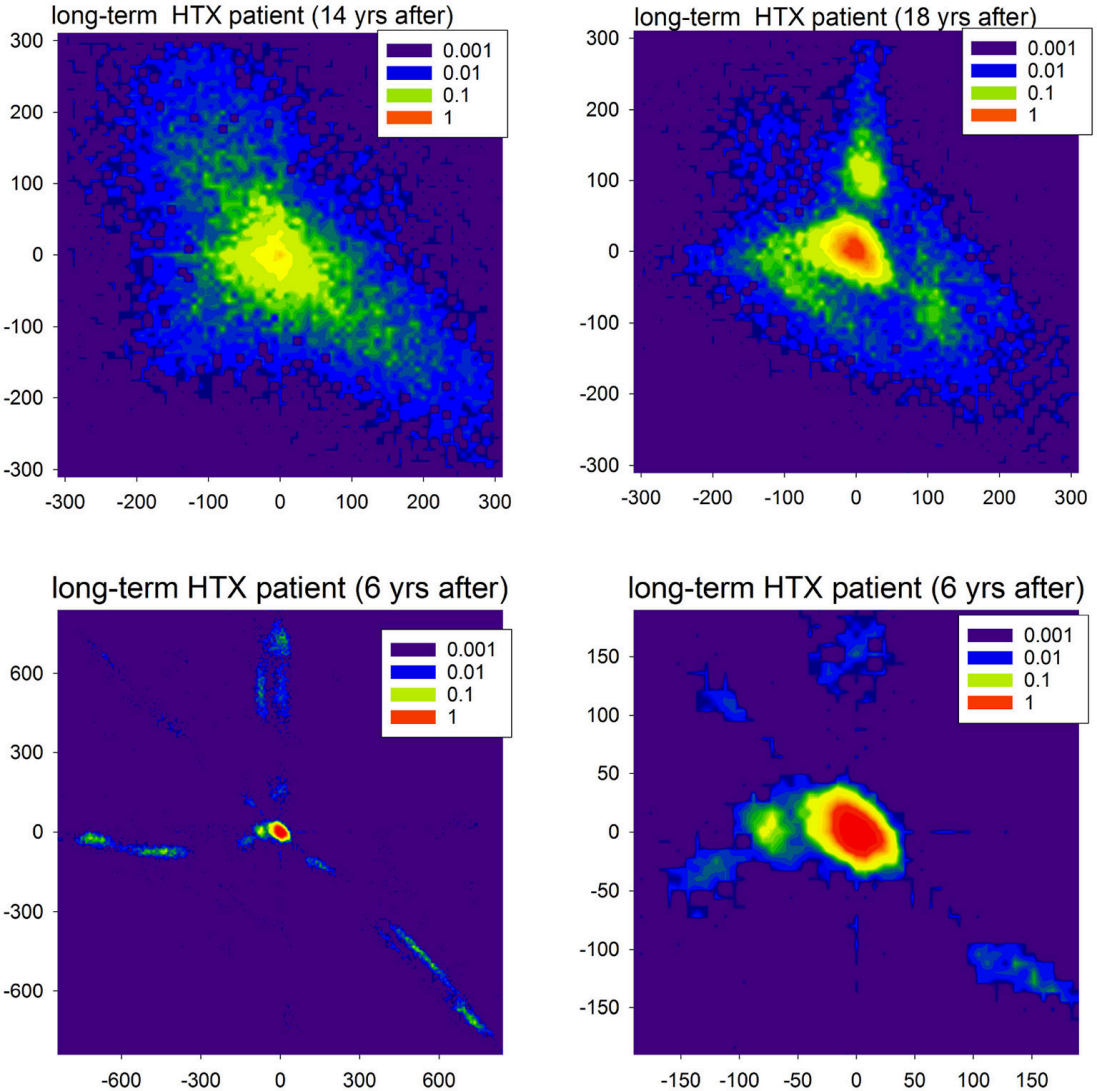
Contour plots of the probability matrix of RR-increments *P*(Δ*RR*_*i*_, Δ*RR*_*j*_) of observing Δ*RR*_*i*_ and Δ*RR*_*j*_ in subsequent time steps for two HTX patients. The top plots display rhythms of the first patient (course free of clinical complications), but at different moments after HTX. The bottom plots display the rhythm of the second patient (course with many clinical complications) twice: full scale and zoomed to present the most probable events.

In the case of most studied HTX patients whose postoperative course went without clinical complications, the probability matrix plots were plain. The patterns were concentrated around (0, 0) ms events which means that the RR-intervals did not change in time with the accuracy of 8 ms, i.e., at the recording resolution. Accelerations or decelerations were of small size, namely less than 50 ms; see Wdowczyk et al. (2018). However, there were HTX patients whose signals with normal RR-intervals presented abnormal patterns. Moreover, heart and/or other diseases have appeared to influence the dynamics of the sinus rhythm. In Figure 9, two of such particular rhythms are presented.

In Figure 9, in the first line, we show matrices obtained from signals recorded in a very long-term HTX patient whose course after the surgery was without complications and other chronic diseases, with the exception of hypertension. The extremely large variability of the patterns of accelerations and decelerations observed 14 years after the HTX surgery, after 4 years transformed into a rhythm concentrated at small size events. However, large departures from this rhythm were evidently present. As that patient underwent the biatrial HTX surgery which preserved the patient's SAN, the observed abnormal variability was hypothesized to be a result of some wide leak of the excitations from their own SAN through the suture line rather than the effect of tissue reinnervation (Wdowczyk et al., 2016).

The course of the patient whose heart rhythm is shown in the second line of Figure 9 was with complications. The patient (after bicaval HTX surgery) suffered from graft vasculopathy, diabetes mellitus, and chronic renal failure. Moreover, there were two events of acute graft rejection. This rhythm, in general, was concentrated at (0, 0) pattern; see the right plot. However, it can be hypothesized that the fatigue of cells, which probably developed in the cardiac tissue, could lead to the number of events with the loss of the excitation front; see the extremal events presented in the left plot. These events can be compared to the missed beats found in simulations. It is important to note that the sophisticated properties of the signals shown in Figure 9 could be misinterpreted as standard features of HRV.

## 5. Modeling Human RR-Interval Variability

The results reported in the previous section were obtained with constant values of all simulation parameters. However, in the case of tissue of a living human, the parameters are presumed to vary in time. In the following, we propose to investigate the effects of *p*_refuse_ variability. From Figure 7, one can learn that by varying this parameter, we can visit different parts of the state-space.

In particular, let us assume that *p*_refuse_ performs a random walk with probability *p*_walk_ in the real valued interval [0, *z*] with some step ε, i.e.,

(8) $$\begin{array}{l} \text{for }\zeta \in U[0,1]\\ p_{\text{refuse}}=\{\begin{array}{ll} p_{\text{refuse}}\pm \varepsilon & \text{if}(\zeta <p_{\text{walk}}\text{ and }p_{\text{refuse}}\pm \varepsilon \in [0,z])\\ p_{\text{refuse}} & \text{if}(\zeta \geq p_{\text{walk}}\text{ or }p_{\text{refuse}}\pm \varepsilon \notin [0,z]) \end{array}\\ \text{where }+\text{ or }-\text{ is chosen at random.} \end{array}$$

In this way, when starting with a normal state we can simulate the combined effects of swings and returns from and to the normal rhythm.

It occurs that from the state with pattern **(A)** of Figure 4, when a high rate of fatigue cells occurs, the wavefront loses its stability. If *p*_refuse_ goes back to smaller values, then many sustained fronts circulating over the cellular network can occur. Such a state leads to an erratic heart rate variability.

**Figure 10:**
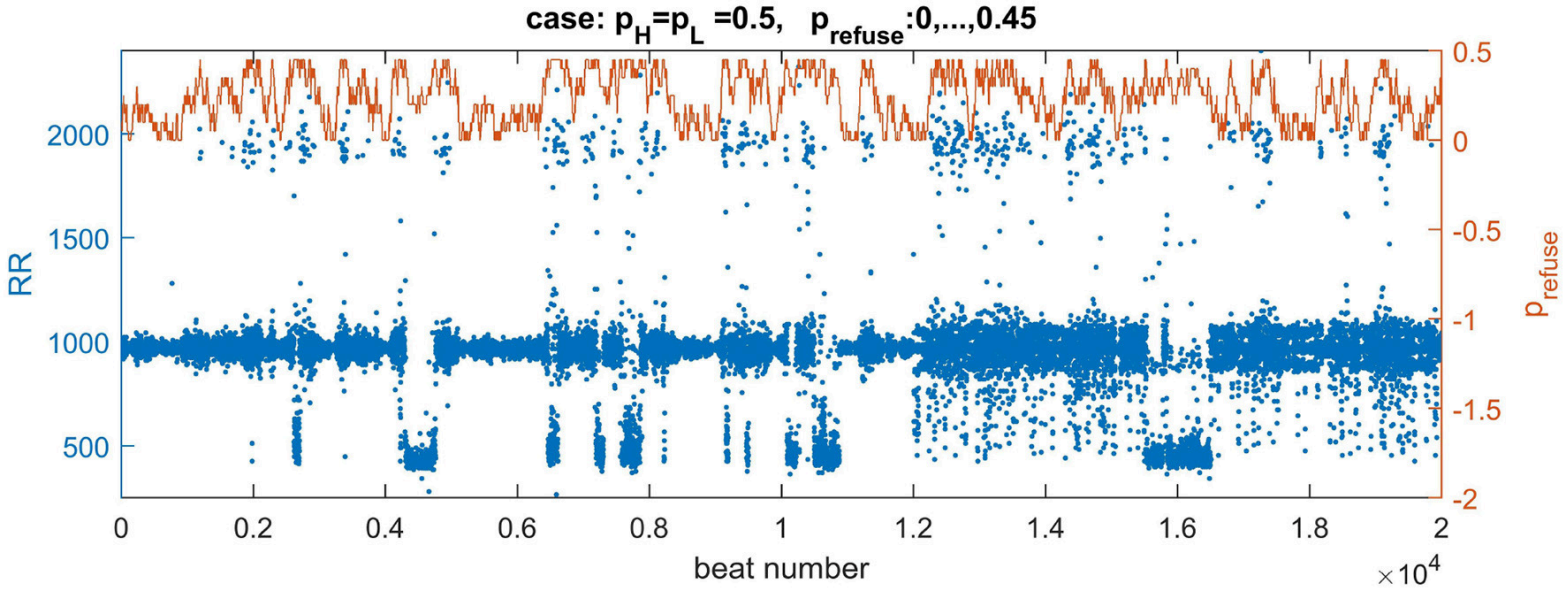
Plots of a walk of *p*_refuse_ performed following Equation 8 (the brown curve) and a resulting signal with RR-intervals (the blue curve). The values of the signal are adjusted (multiplied by 7) to correspond better to the range of real signals. The right axis serves a description for *p*_refuse_. The curve of *p*_refuse_ is plotted above the RR-signal to provide insights how the level of *p*_refuse_ influences both the value and variability of RR-intervals.

Depending on the density of transversal connections, the switch from the normal state to the lost front state occurs at a different *z* value. Based on our results, see the table of states in Figure 7, we have found that in the case of transversal connections established with probability *p*_*H*_ = *p*_*L*_ = 0.5, the swing out and back to the normal state can be achieved when this limit value is set to *z* = 0.45. Additionally, to reduce the number of missed beats, we assume AVN excitation if at least two AVN cells are simultaneously excited. In Figure 10, one can find a plot of typical time signal obtained from an experiment when a system evolved with *p*_refuse_ which performed a random walk described in (8) with *p*_walk_ = 0.001 and ε = 0.05.

In Figure 10 we may observe directly how transitions in *p*_refuse_ act on the system evolving with the normal rhythm. The small levels of *p*_refuse_ slightly influence the normal rhythm. At mid levels of *p*_refuse_ some beats are missed. Then with increasing *p*_refuse_ the system produces a large spectrum of beats: very short and very long ones.

**Figure 11:**
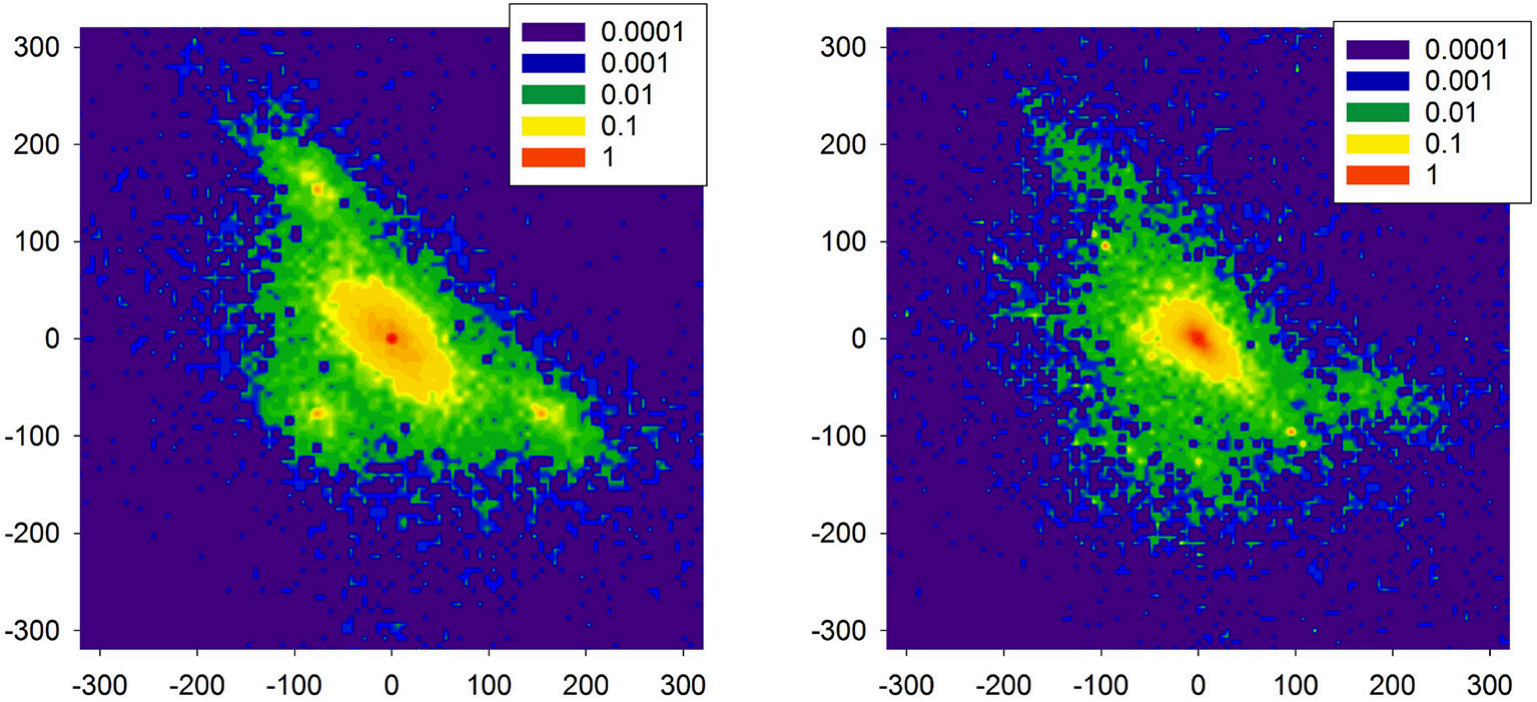
Probability of transition matrix obtained from simulations (20000 RR-increments), log-plots. The left plot refers to the time series shown in Figure 10. The right plot starting point was characterized by the high presence of the native SAN.

In Figure 11, distributions are shown of the probability of increments *P*(Δ*RR*_*i*_, Δ*RR*_*j*_) to illustrate two increments Δ*RR*_*i*_ and Δ*RR*_*j*_ in a sequence in a time series obtained from the model simulations. The left plot reports properties of the signal presented in Figure 10. One can observe the presence of the peaks which are different from (0, 0). These peaks are related to switches to the SAN arrhythmia state.

As the model is dedicated to the HTX patients, therefore, we also investigated one special aspect of the right atrium architecture which is characteristic for an HTX patient after so-called biatrial surgery. In such a case, the right atrium consists of two parts: the donor part with the donor pacemaker, and the native part in which the native pacemaker acts; for details, see Figure A2 in Appendix: Model of a biatrial HTX patient. These two parts are separated by the suture line. However, with time passing this line can leak the native SAN activation to the graft. The right plot of Figure 11 shows the distribution obtained from simulations with this special architecture of right atrium, in a case when the suture line was leaking. Additionally, to enrich the interaction structure, links between cells were locally modified (see Makowiec, 2005 for description of the modification procedure). In consequence, the native SAN activity with slower beats actively perturbed signals issued by the donor SAN. One observes a broad variety of large size RR-increments.

Comparing the two plots of Figure 11 with the plots obtained from the real HTX patients presented in Figure 9, one may hypothesize that our modeling can provide an explanation for the observed dynamical landscape of the heart rhythm of the long-term HTX patient. Note that both arrhythmias: the SAN arrhythmia and the native SAN rhythm, are normal rhythm perturbations which are hardly recognizable from the ECG, even by a highly qualified cardiologist.

## 6. Discussion and Summary

Progress in our understanding of multi-component, multi-layered and spatially complex composite systems has created an excellent foundation for exploring and evaluating the structural and dynamic characteristics of real systems based on experimental data (Bashan et al., 2012; Havlin et al., 2012; Müller et al., 2016).

In the model we assumed that each AVN excitation propagated further over the remaining cardiac tissue smoothly, resulting in a proper ventricular contraction. Based on this we could describe the rhythm of heart contractions by time intervals between subsequent AVN excitations. Subsequently, the standard heart rhythm analysis might be applied which means that computer model series might be directly verified by clinical observations. However, only the heart rhythm of HTX patients could be of interest to us because the model does not consider autonomic system regulation. There are also other evident model limitations. We did not consider the role of cardiac fibroblasts in the maintenance of the extracellular matrix which provides the framework for cardiac tissue and is crucial in the passive mechanical properties. However, the global effect of the fibroblasts, namely, the growing fibrosis, was in the center of our interest. Also the AVN has been modeled in a simplified way. The few cells, which we considered as the AVN, represent merely the transition region to the node. Therefore, the role of the AVN has been limited to detecting of the arrival of a wave-type signal. In particular, we have considered that 1/3 of the AVN cells should be excited to result in further signal transmission. However, in the simulations presented in section 5, we assumed that simultaneous excitation of two of AVN cells is enough to excite the AVN. This way, the ratio 1/3 has become a non-named parameter of a model, which role could be investigated in further research. Finally, we have to underline that the partition of the space of limit states shown in Figure 7 depends strongly on the definition of the normal rhythm given in Equation (7). The classification of limit states, presented in Figure 5, is based on RR-intervals variability and only discussed above excitability of the AVN.

Though our approach is dealing with a simplistic representation of the complex architecture of the human right atrium, as we could see, it was sufficient to determine whether the system is critical. Namely, we could point at the model parameters, a slight change in which could result in a strong perturbation, or even in the loss of the rhythm of heartbeats. As these parameters are physiologically grounded, we could provide observations on a rather large variety of very general cardiac electrophysiology relations and/or their aspects. In particular:

(i) Influence of the increase of collagen deposits on the impaired conduction of the impulse which was mimicked by the decrease in density of horizontal *p*_*H*_ and lateral connections *p*_*L*_. We have found this dependence critical. For high levels of *p*_*H*_ and *p*_*L*_, the development of the normal rhythm is almost sure. Then the probability that the normal rhythm occurs is switched to zero in a rather narrow interval of the model parameters.
(ii) An impairment or fatigue of the individual cells caused by the cardiac or other diseases, so the impact of individual cell performance was studied in two aspects. The first aspect, revealing the heterogeneity of APD length, wassimulated with the parameter *r*_noise_ by considering a randomspread of APD value. The second aspect—the dysfunctionof a cell, was mimicked by variation with the parameter*p*_refuse_ describing the level of the refuse of a cell to follow the excitation. It is noticeable that we have admittedthe stabilizing role of the stochasticity when these twoaspects have low levels. But after crossing some thresholdswe observe the critical breakdown of the wavefront.
(iii) The important characteristic of 2D propagation is a dependency of velocity on curvature (Clayton et al., 2011). As it was expected, the convex fronts developed at a higher density of transversal connections propagated faster than the fronts observed in cases when the transversal connections were rare.

The architecture proposed by us can be compared to a model considered in Podziemski and Zebrowski (2013) or Kharche et al. (2017). However, the localization of the SAN in our model enabled observation of re-entry around the SAN. Also, our model can be seen as motivated by the ideas of the Christensen et al. (2015) model. Similarly to them, we considered automata which were always vertically connected (*p*_*V*_ = 1). However, additionally, we included randomly established lateral connections. We assumed also that all cells were dysfunctional, i.e., each automaton with the probability *p*_refuse_could refuse the excitation. However, the main novelty of our model is that it has been rewritten into the hybrid automaton formalism which underlines and provides a direct link to the relations between a discrete transitions graph and continuous dynamics of processes involved in a myocyte excitation. Therefore further model development is straightforward. The possible extensions include effects of limits in pathways between the SAN and atrium, local areas of fibrosis, further diversity of cells.

There is clinical evidence that continuous models might fail to capture the specificity of arrhythmia (Gokhale et al., 2016, 2017). This fact promotes discrete modeling (Trayanova, 2014). The hybrid automata appear to be the perfect modeling tool in revealing both intracellular and intercellular biological relations (Ye et al., 2006; Bartocci et al., 2010; Li et al., 2014; Christensen et al., 2015). The computational efficiency of these models makes it possible to test various predictions about heart electrophysiological behavior with relatively low computational cost. The model proposed here is an efficient real-time model. The enumerated specification provides an easy path for the further development of the model.

The model is especially dedicated to the heart electrophysiology after HTX, i.e., to humans with a strongly reduced presence of the autonomic nervous system. Because of this, we were able to link directly the heart rate variability of real people to results obtained in simulations. In particular, we have shown that the evidence of high levels of heartbeat variability in the absence of regulation served by the autonomic nervous system may be related to an increase in cellular fatigue, which possibly results from the remodeling of cardiac tissue caused by immunosuppressive drugs or the active presence of the native SAN.

## Author Contributions

DM and ZRS conceived and designed the study. JW provided the medical data. DM designed and wrote the computer program, performed the simulations and data analysis. All the authors interpreted the simulation results. DM drafted the manuscript. All the authors edited and then read and approved the final manuscript.

### Conflict of Interest Statement

The authors declare that the research was conducted in the absence of any commercial or financial relationships that could be construed as a potential conflict of interest.
