# Supplementary material for: Heart Rhythm Insights Into Structural Remodeling in Atrial Tissue: Timed Automata Approach
Source: Front Physiol. 2019 Jan 14;9:1859. doi: 10.3389/fphys.2018.01859 (PMC6340163; doi:10.3389/fphys.2018.01859)
Supplement: Supplementary file 1 [file Presentation_1.pdf]

Heart rhythm insights into structural remodeling in atrial tissue: timed automata approach

Danuta Makowiec [1*], Joanna Wdowczyk [2] and Zbigniew R. Struzik [3,4]

[1] *Institute of Theoretical Physics and Astrophysics, University of Gdańsk, Poland*
[2] *1st Department of Cardiology, Medical University of Gdańsk, Poland*
[3] *RIKEN Brain Science Institute, Japan*
[4] *University of Tokyo, Graduate School of Education, Japan*

Correspondence*:
Danuta Makowiec
danuta.makowiec@ug.edu.pl

APPENDIX

Simulation specification

The conditions and values for intrinsic cellular dynamics were selected to best fit the physiological facts. For example, under normal conditions, only one wavefront is observed propagating in the real atrium. Therefore, the SAN period $T = f_{\text{SAN}} + r_{\text{SAN}} + a_{\text{SAN}}$ should match the length of the diagonal of the lattice $L \times L$. Then, in accordance with the known properties of the AP in the myocytes, see Fig. 2 of the Article, the values of f, r, a were assigned as it is shown in Fig. 1.

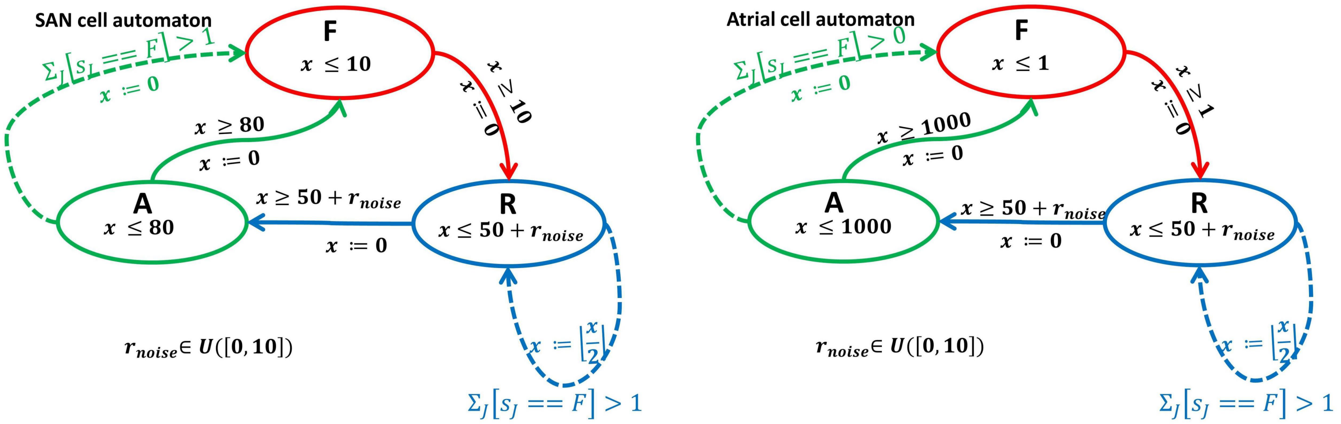

Figure 1. The specification of the timed automata, a SAN cell (left) and atrial cell (right), used in simulations as the transition graphs.

The results shown in the paper were obtained with the following parameters and conditions kept fixed:

(1) $L = 120$;

(2) SAN consists of 5×15 cells located at (10, 10);

(3) AVN consists of eight cells located at $(100, 120)$: 2, 3, 3 neighboring cells from subsequent rows; AVN is excited if three of eight AVN cells are simultaneously excited;

(4) intrinsic dynamics of the atrial cell: $f = 1, r_0 = 50, a = 1000$ and of the SAN cell: $f_{\text{SAN}} = 10, r_{0,\text{SAN}} = 50, a_{\text{SAN}} = 80$, see graphs of timed automata in Fig. 1

(5) atrial intercellular are connections: $p_V = 1$, $p_H = p_L$, nodal intercellular are connections: $p_V = p_H = p_L$.

The effects of the following changes in the model parameters were observed:

(a) $p_H = 0.05, 0.10, 0.15, 0.20, 0.30, 0.40, 0.50, 0.65.0.80$, the same for nodal and atrial connections;

(b) $p_{\text{refuse}} = 0.00, 0.05, 0.10, 0.20, 0.30, 0.40, 0.50, 0.60$, the same for nodal and atrial connections;

(c) $r_{\text{noise}} = 0$ or 10;

Each parameter setting was simulated a hundred times. After an initial one thousand steps allowed for the system stabilization, the state classification was performed according to the number of wavefronts reaching the AVN during the next 3000 steps. The time between two successive arrivals of fronts is denoted RR. The classification of Fig. 5 of the Article provides the following conditions on RR:

normal rhythm if $\min RR > 120$ and $19, \ldots, 24$ beats were counted;

lost normal rhythm if $\min RR > 120$ and $8, \ldots, 18$ beats were counted;

SAN arrhythmia if $\min RR$ and $\max RR \leq 90$;

lost arrhythmia if $\min RR \leq 120$ and $8, \ldots, 18$ beats were counted;

dead rhythm if the AVN was excited less than eight times.

Model of a biatrial HTX patient

The model of a HTX patient after biatria surgery reconstructs the specific architecture of the atria, see Fig. 2, namely existence of two SANs: the donor SAN and the native SAN. In particular, we simulated the

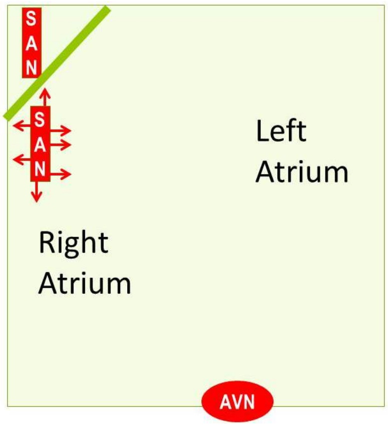

Figure 2. The architecture of the atria used in simulations of a HTX patient after biatria surgery

model with the following parameter settings:

(1) $L = 150$, the suture line starts at (0,45) and ends at (45,0) and consists of 5 parallel lines of non-interacting cells;

(2) each SAN consists of 5×15 cells located at (10, 50) in case of the donor SAN, or at (10,10) in case of the native SAN;

(3) intrinsic dynamics of native SAN automaton is assumed as slow $f = 10, r_0 = 100, a = 100$;

Additionally, to get the effect of leaks of the native SAN excitation through the suture line, the links between cells were locally rewired with probability 0.1, see Makowiec (2005) for description of the local rewiring procedure.

Model implementation and statistical estimates

For the model implementation, we used mixed programming techniques. The basic software is in C++, of which real-time window implementation in Windows, is accessible from Makowiec (2018). The statistics of the state classification were calculated with MATLAB R2016b (The MathWorks Inc.) and Sigma Plot 13.0 (Systat Software, Inc).

REFERENCES

Makowiec, D. (2005). Evolving network – simulation study. *The European Physical Journal B - Condensed Matter and Complex Systems* 48, 547–555. doi:10.1140/epjb/e2006-00008-2

[Dataset] Makowiec, D. (2018). Atrium: timed automata model of human right atrium electrophysiology. https://github.com/DanutaMakowiec/atrium
